# Clinical parameters of implants placed in healed sites using flapped and flapless techniques: A systematic review

**DOI:** 10.4317/medoral.21897

**Published:** 2017-08-16

**Authors:** Oscar Llamas-Monteagudo, Paula Girbés-Ballester, José Viña-Almunia, David Peñarrocha-Oltra, Miguel Peñarrocha-Diago

**Affiliations:** 1 DDS, Resident of the Master in Oral Surgery and Implant Dentistry, Stomatology Department, Faculty of Medicine and Dentistry, University of Valencia, Spain; 2 DDS, PhD, Collaborating Professor of the Master in Oral Surgery and Implant Dentistry, Stomatology Department, Faculty of Medicine and Dentistry, University of Valencia, Spain; 3 DDS, PhD Assistant Doctor Professor of Oral Surgery, Oral Surgery and Implantology Unit, Stomatology Department, Faculty of Medicine and Dentistry, University of Valencia, Spain; 4 MD, DDS, PhD, Chairman of Oral Surgery, Stomatology Department, Faculty of Medicine and Dentistry, University of Valencia, Spain

## Abstract

**Background:**

Dental implant placement using flapless surgery is a minimally invasive technique that improves blood supply compared with flapped surgery. However, the flapless technique does not provide access to allow bone regeneration.

**Objectives:**

The aim of this systematic review was to evaluate the clinical parameters following implant surgery in healed sites, using two procedures: flapped vs. flapless surgery.

**Material and Methods:**

A detailed electronic search was carried out in the PubMed/Medline, Embase and Cochrane Library databases. The focused question was, “How do flapped and flapless surgical techniques affect the clinical parameters of dental implants placed in healed sites?”. All the studies included with a prospective controlled design were considered separately, depending on whether they had been conducted on animals or humans. The following data were recorded in all the included studies: number of implants, failures, location (maxilla, mandible), type of rehabilitation (partial or single), follow-up and flap design. The variables selected for comparison in the animal studies were the following: flap design, gingival index, mucosal height, recession and probing pocket depth. In humans studies the variables were as follows: flap design, plaque index, gingival index, recession, probing pocket depth, papilla index and keratinized gingiva.

**Results:**

Ten studies were included, six were experimental studies and four were clinical studies. Studies in animals showed better results using the flapless technique in the parameters analyzed. There is no consensus in the clinical parameters analyzed in human studies, but there is a trend to better results using flapless approach.

**Conclusions:**

The animal studies included in the present review show that implants placed in healed sites with a flapless approach have better clinical parameters than the flapped procedure in a short-term follow-up. In human studies, there is no consensus about which technique offer better results in terms of clinical parameters. Therefore, more research in humans is required in order to overcome the limitations and contrast these results.

** Key words:** Clinical parameters, gingival recession, probing depth, dental implants, flap, flapless.

## Introduction

Flapped implant placement involves exposure of the alveolar ridge using a full-thickness mucoperiosteal flap, placement of the implant and suture of the flap (1,2). This conventional technique facilitates visibility and access at the operating site, ensuring that some anatomical landmarks are clearly identified and protected (3), while it provides the possibility of regenerating bone fenestration and dehiscences and resolves other complications. Flapped surgery is considered advantageous in the aesthetic zone since flaps can be repositioned to desired locations (4) and it also makes it possible to prevent ingrowth of gingival tissue between the implant and the bone (5). However, reflection of the mucoperiosteal flap compromises the vascular supply of bone (6), which may lead to crestal bone loss and long-term aesthetic complications (4,7,8). The correlation between flap elevation and bone loss (9,10-13) resulted in the introduction of minimally invasive or flapless techniques in the late 1970s by Ledermann (14). Flapless implant placement is usually performed by minimum incision (15-17), perforation with the drill through the soft tissues (16,18,19), or soft tissue removal using a tissue punch (16,20,21). Several studies have shown that flapless implant surgery allows a reduction in surgical time, maintenance of both soft and hard tissues, decreased postoperative bleeding, faster recovery and is more comfortable for the patient (5,15,18,22-24).

Becker *et al.* (18) evaluated implant placement using the flapless technique after two years; the results showed minimal changes in crestal bone level, probing depth and inflammation, demonstrating that the flapless technique is a predictable procedure. Similar results were reported by Jeong *et al.* (15) who reported that the peri-implant bone height was greater at flapless sites. Another study by Lee *et al.* (25) investigated the effects of flapless implant placement on soft tissue profiles in 44 patients, and their outcomes indicated that the flapless technique is superior to the flap implant procedure for maintaining the original mucosal profile around implants. On the other hand, significant disadvantages of flapless placement include the inability to visualize anatomic landmarks and vital structures, the potential for thermal osseous damage from the obstructed external irrigation, the inability to contour bone morphology, the increased risk of implant misplacement in relation to angulation or depth, keratinized gingival tissue loss and the inability to manipulate soft tissues around an emerging implant (26). Despite the drawbacks of flapless surgery, currently with the help of 3-dimensional imaging techniques and computer-guided implant planning, implants can be placed more accurately with less risk (15,27,28).

At present, there are only two systematic reviews that evaluate peri-implant bone loss in flapless vs. flapped surgery in dental implants (29,30). The publication by Vohra *et al.* (29) on ten clinical studies, concluded that marginal bone loss around dental implants placed in healed sites is comparable, although implants in four studies showed significantly less crestal bone loss in the flapless group. To the current authors’ knowledge from indexed literature, there are few studies available that provide data about other clinical parameters comparing both techniques. The objective of this systematic review was to evaluate the clinical parameter changes following implant surgery, using two procedures: flapped vs. flapless surgery.

## Material and Methods

A systematic review was carried out in accordance with the PRISMA (31) (Preferred Reporting Items for Systematic Reviews and Meta-Analyses) recommendations.

The focused question was, “How do flapped and flapless surgical techniques affect the clinical parameters around dental implants placed in healed sites?” 

- Search strategy 

To identify the relevant studies, a detailed electronic search was carried out in PubMed/Medline, Embase and Cochrane Library databases using different combinations of the following key words: “clinical parameters”; “gingival recession”; “probing depth”; “dental implants”; “open surgery”; “surgical flaps”; “flap” and “flapless”. The following limits were applied: studies published in dental journals and in English. The search was updated in May 2016. All studies, without restriction on the publication date, were analyzed.

- Study selection criteria

The following eligibility inclusion criteria were applied: 1) Prospective controlled study design comparing clinical implant para-meters using flap and flapless techniques in humans or animals; 2) Implants placed in healed sites; 3) Studies involving more than ten implants in each group comparing at least one of the following clinical parameters: gingival index, plaque index, probing pocket depth, recession, mucosa height and inflammation; 4) Studies had to specify the survival rate; 5) and a minimum follow-up of 1 week.

- Exclusion criteria were the following: 1) Case reports; 2) Systematic reviews or technical notes; 3) Immediate implant placement technique; 4) Implants with simultaneous bone regeneration.

Two reviewers independently assessed the titles of all the articles. If the abstract did not provide sufficient information for a definite decision on inclusion or exclusion, the full article was obtained and reviewed before the final decision was made. In the event of disagreement, discussions were held until consensus was reached; however, if the reviewers continued to disagree, a third reviewer was consulted.

- Assessment of risk of bias in included studies

The risk of bias assessment of the included studies was undertaken independently and in duplicate by at least two review authors as part of the data extraction process. The assessment was conducted using the recommended approach for assessing risk of bias in human studies included in Cochrane reviews (32) and also using SYRCLE´s Risk of Bias tool for animal intervention studies (33).

The Risk of Bias tool for human studies is a two-part tool, addressing the seven specific domains (namely sequence generation, allocation concealment, blinding of participants and personnel, blinding of outcome assessment, incomplete outcome data, selective outcome reporting and ’other issues’). Each domain includes one specific entry in a ’Risk of bias’ table. Within each entry, the first part of the tool involves describing what was reported to have happened in the study. The second part of the tool involves assigning a judgement relating to the risk of bias for that entry. On the other hand, the SYRCLE´s Risk of Bias tool contains 10 entries. These entries are related to selection bias, performance bias, detection bias, attrition bias, reporting bias and other biases. Half these items are in agreement with the items in the Cochrane Risk of Bias tool.

- Data synthesis and analysis

The studies were considered separately, depending on whether they had been conducted on animals or humans. The following data were recorded in all the studies: number of implants, failures, location (maxilla, mandible), type of rehabilitation (partial or single), follow-up and flap design. The variables selected for comparison in the animal studies were the following: gingival index (GI), mucosal height (epithelial attachment + connective tissue height), recession and probing depth (PD). In human studies, the data analyzed were: plaque index (PI), gingival index (GI), gingival recession, probing depth (PD), papilla index (PPI), keratinized gingiva (KG).

## Results and Discussion

- Study selection and description

**Figure 1:**
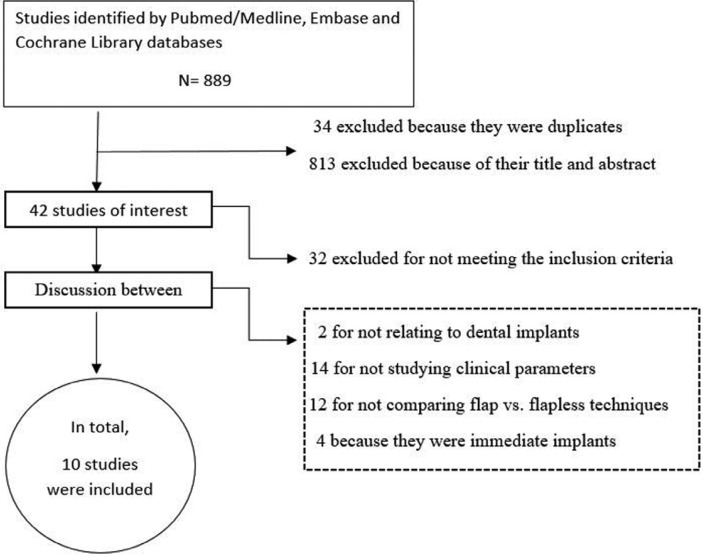
Flow chart for the systematic review.

The first stage of the search identified a total of 889 articles. Of these, 34 were duplicates and were excluded. On critical reading of the title and abstract, 813 articles were excluded because they did not answer the research question, leaving a total of 42 articles. On reading the full text of these articles, 32 were excluded because of the following reasons: 2 for not relating to dental implants, 14 for not studying clinical parameters, 12 for not comparing flap vs. flapless techniques, and 4 for being immediate implants. The resulting 10 studies were included (Fig. 1) and are detailed separately in humans and animals ( Table 1, Table 1 continue, Table 2,  Table 2 continue).

**Table 1:**
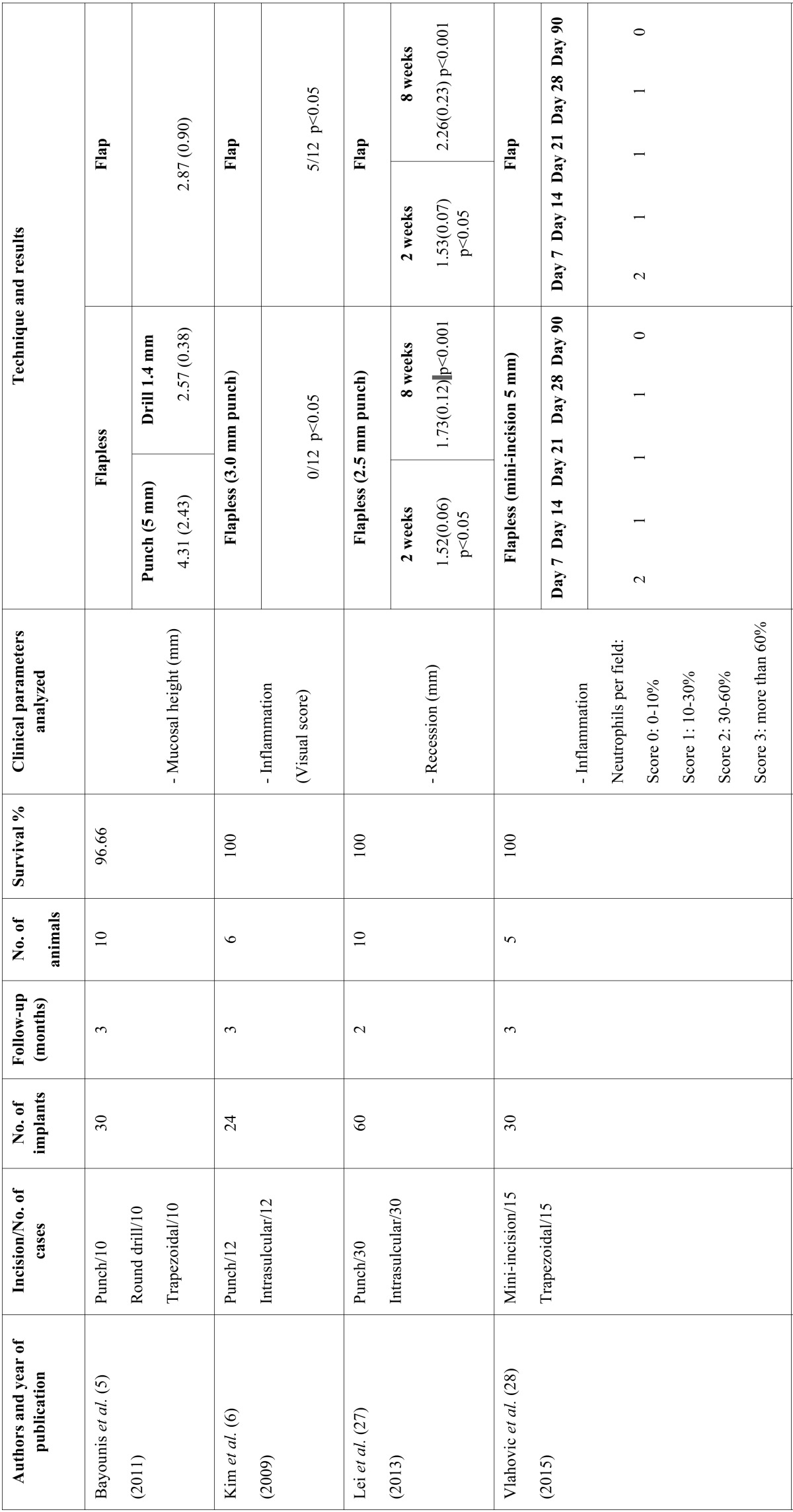
General data recorded and clinical parameters in animal studies.

**Table 1 continue:**
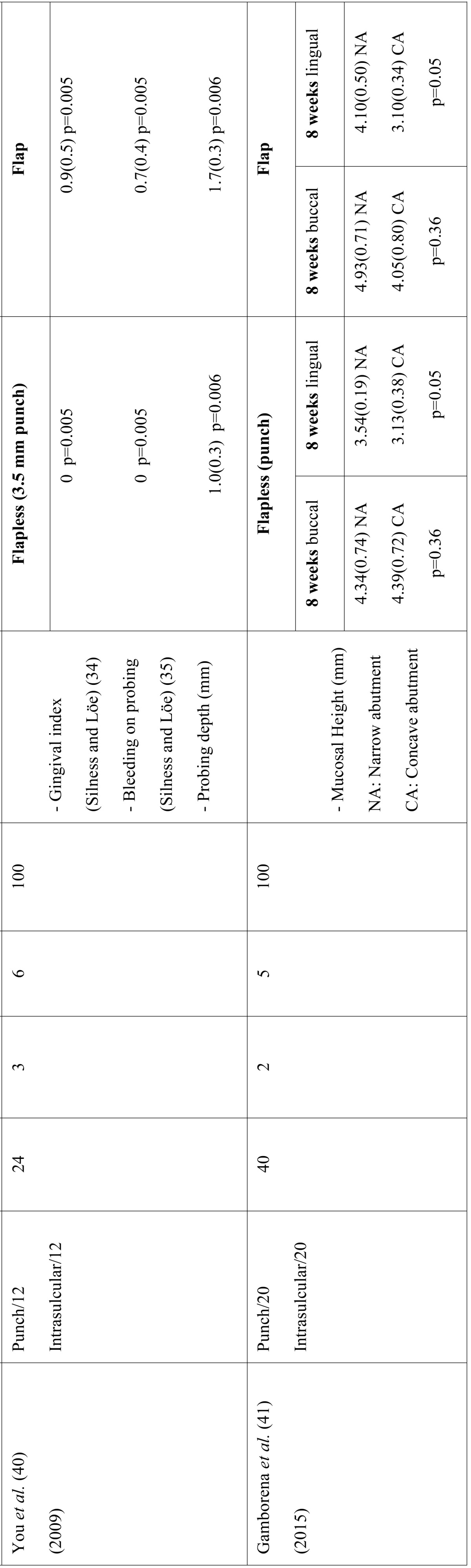
General data recorded and clinical parameters in animal studies.

**Table 2:**
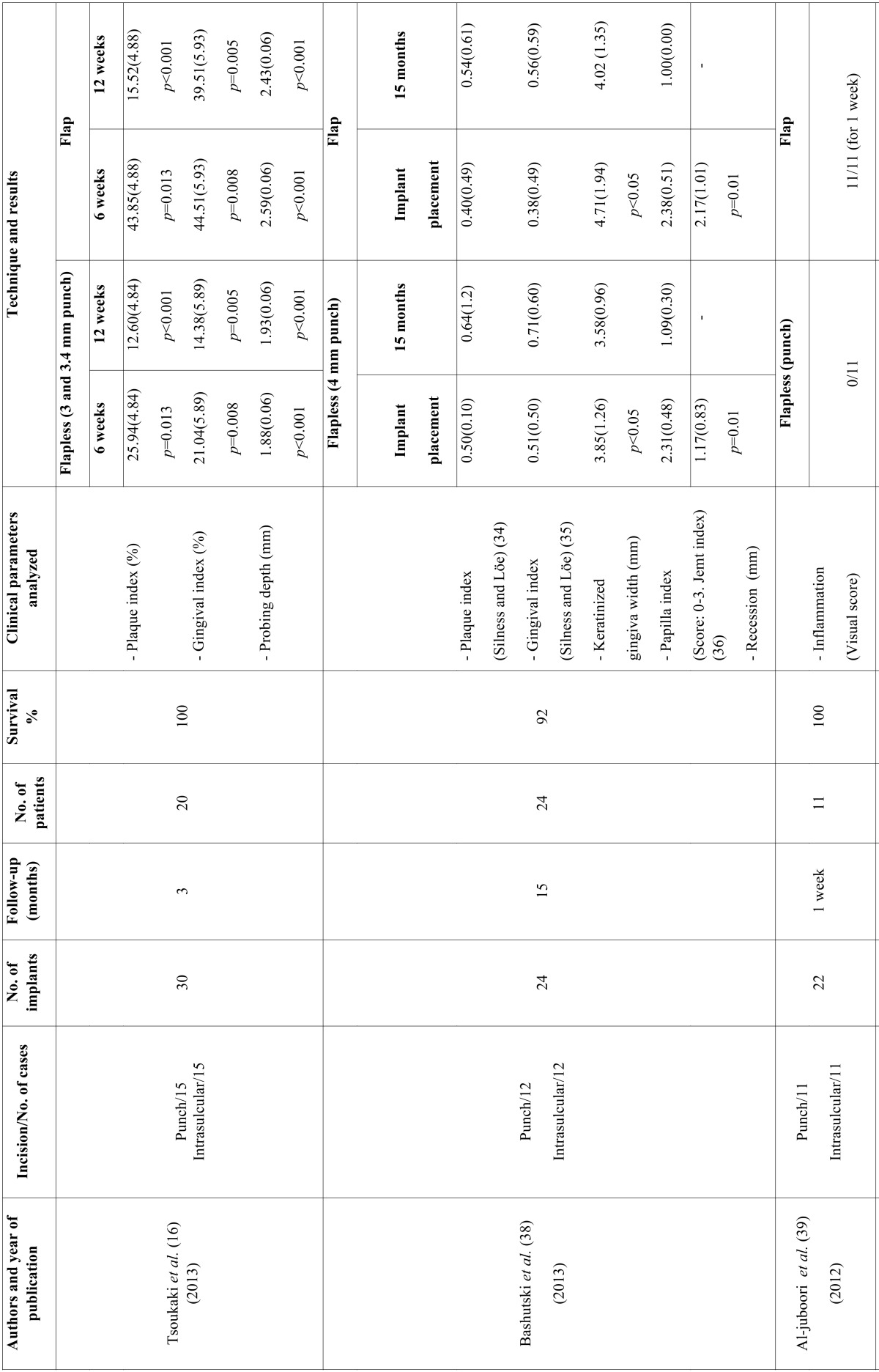
General data recorded and clinical parameters in human studies.

**Table 2 continue:**
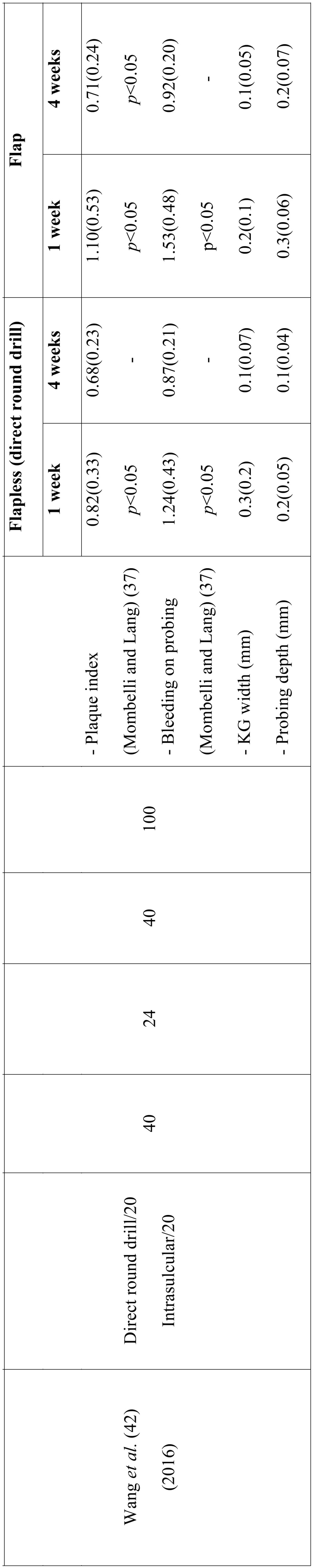
General data recorded and clinical parameters in human studies.

The number of implants placed ranged from 22 to 60 with a total of 334 implants placed in the studies that were included and 3 implant failures were recorded. All implants were placed to provide unitary or partial rehabilitation. In all the included studies, except for the one performed by Bashutski *et al.* (38), the location of implants was in the mandible. The follow-up period ranged from 8 weeks to 24 months, except the study by Al-juboori *et al.* (39) which only had a 1-week follow-up.

Different flap designs were performed; in eight studies (6,16,27,38-42) a midcrestal flap with intrasulcular incision was performed, and in two studies (5,28) a trapezoidal flap was used.

Flapless surgery was as follows: in eight studies (6,16,27,38-42), implant placement was made with a soft tissue punch protocol. In two studies (5,42), no soft tissue preparation was used, penetrating through the mucosa without incision. In one study (28), a flapless technique was carried out with a mini-incision of 5 mm horizontal to the alveolar crest.

- Overall risk of bias

It is evident that is not possible to blind operators and patients to the allocated intervention in a surgical trial. Moreover, animal intervention studies differ from randomized clinical trials in many aspects, as the methodology for Systematic Reviews of clinical trials needs to be adapted and optimized for animal intervention studies. Therefore there is a situation in that both a well-designed trial in which everything is described in detail, and a poorly reported trial where authors simply did not describe the methodological procedures adopted to minimise bias, are both likely to be rated as being at unclear risk of bias.

For this reason, the results from the risk of bias summary tables ( Table 3, Table 4) should be interpreted with caution.

**Table 3:**
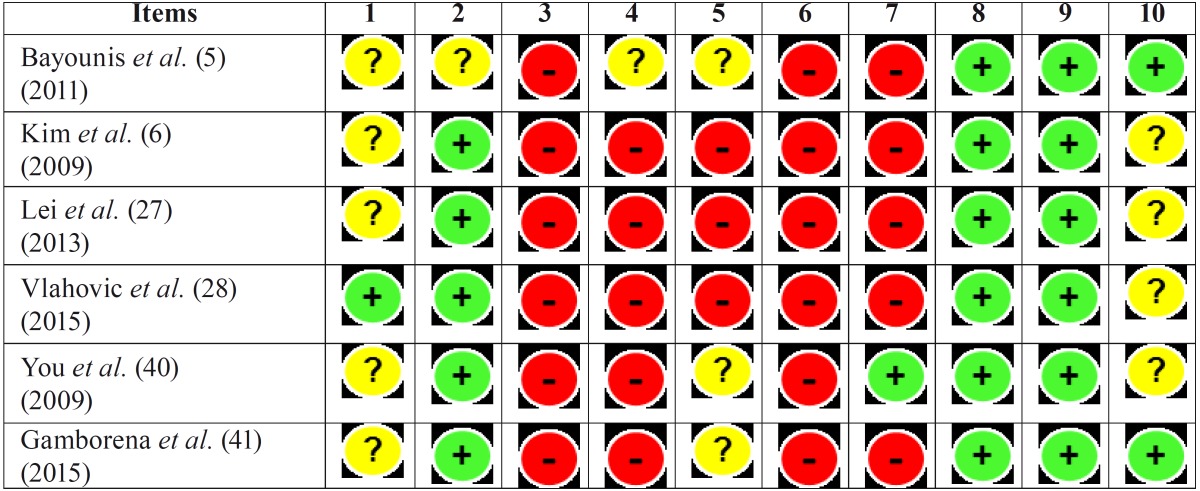
Risk of bias summary in animals studies.

**Table 4:**
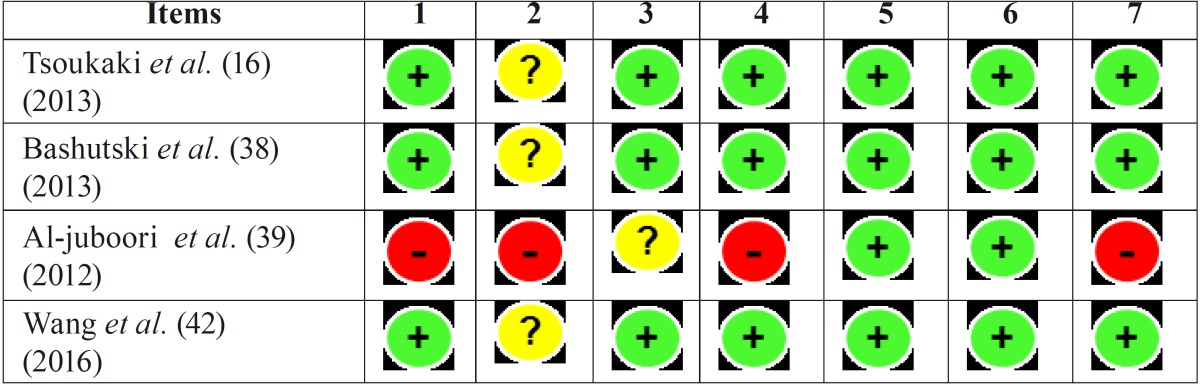
Risk of bias summary in humans studies.

- Animal studies

The GI was evaluated in one study (40), which demonstrated that flapless surgery involved less postoperative inflammation than the flap approach (score of 0 and 0.9, respectively).

The mucosal height was assessed in two studies (40,41). The study by You *et al.* (40) showed that mucosal height was thicker at flap than flapless sites, with measurements of 3.5 mm vs. 2.2 mm, respectively. In the other study (41), mucosal height was significantly higher at lingual sites with flapless surgery, while at buccal sites there were no significant differences. The epithelial attachment was analysed in three studies (5,40,41). Bayounis *et al.* (5) performed a study comparing three groups. They showed that epithelial attachment was higher using flapless surgery with the punch technique than flap surgery (mean 2.27 and 1.38 mm), but the lower level of epithelial attachment (1.1 mm) was found with transmucosal implant placement (without punch). Different results were reported in another study (38), where the height of the epithelial attachment was statistically significant (2.2 mm for the flap and 1.2 mm for the flapless group). On the other hand, in the study by Wenzel *et al.* (41) no significant differences were observed between groups for the apical extension of the epithelial attachment.

The recession was only analysed in one study (27), and there was no significant difference between the flap and flapless groups at the second week. Although after 4 and 8 weeks, recession was less pronounced in the flapless procedure, providing evidence that flapless surgery caused minor epithelial contraction, and therefore better aesthetic outcomes of implants.

The PD was assessed in one study (40). PD was significantly greater in the flap group than in the flapless group (mean 1.7 mm and 1.0 mm, respectively).

Inflammation was evaluated by Kim *et al.* (6), who showed that soft tissue around all implants in flapless sites appeared to be free from signs of inflammation, whereas it was red and oedematous in 5 of 12 implants in flap sites. These results demonstrated that flapless implant placement reduced peri-implant soft tissue inflammation, leading to faster recovery.

- Human studies

The PI was assessed in three studies (16,38,42). In two of them (16,38) flapped implants exhibited a higher PI compared with flapless implants, although these levels decreased at 3 months and this difference was no longer statistically significant at 15 months. Similar outcomes were found in another study (42) with statistically significant differences between two groups until 1 and 2 weeks post-surgery.

The GI was evaluated in four studies (16,38,39,42). Two (16,38) showed significantly higher GI values in the flap compared with flapless groups at 3 and 9 months. The other two studies (39,42) initially presented similar results although there was a decrease at 1 and 4 weeks, respectively.

The recession and the PPI were only assessed in the study by Bashutski *et al.* (38). There were no significant differences in recession between groups at any time point. On the other hand, patients who received implant placement using a flap approach had an initial decrease in their PPI, whereas the flapless group had a significant increase in their PPI during 6 months. The PPI increased over time in both groups, although the flapless group had a significantly larger increase at 6 and 9 months. No differences in the PPI were noted between flap and flapless groups in patients with thin biotypes. By contrast, patients with a thick biotype who received flapless implant placement had a trend towards greater papilla fill than the flap group at 9 months after placement. This difference was no longer significant at 15 months.

The KG width was assessed in two studies (38,42). One study (38) showed statistically significant differences in the width of KG between the flap and flapless groups, (mean of 0.86 mm in the flap group). Both groups had a decrease in the amount of KG, although the flap group experienced a greater loss of KG over time. In the other study (42), the average of KG in the flapless and flapped groups was 4.2 and 4.5 mm, respectively before treatment, and decreased to 3.7 mm in the flapless and 4.0 mm in the flapped group at the day of the abutment connection. However, the mean KG remained stable at the 24-months follow-up.

The PD was analysed in two studies (16,42). In both studies, the PD was significantly higher in the flap group compared with the flapless group. Specifically, in the study by Wang *et al.* (42), the PD increased in the flap group on the day of abutment connection compared to 4 weeks post-surgery, however, it proved stable at the following visits and no difference was detected.

- Implications for practice

The results of the present study suggest that implants placed in healed sites using flapless technique undergo better clinical parameters around implants compared to those placed using conventional surgical flap procedures. An explanation of these results may be derived from the fact that flapless surgery allows minimum disruption of peri-implant tissues. In addition, it also allows to preserve circulation of the peri-implant tissues and accelerate recuperation, allowing the patient to resume normal oral hygiene procedures immediately after surgery.

However, some results of the present study show no significant differences between both techniques. There is still insufficient evidence regarding a potential increased risk of complications/failures using a flapless approach. For this reason, the main draw-backs of this technique, such as limited bone width, lack of keratinized tissues, the difficulty in assessing the implants’rough surface is totally surrounded by bone, should make the clinicians select patients for flapless implant placement with a great deal of caution in relation to their own clinical skills and experience in order to increase the benefits in the implant placement procedure.

## Conclusions

Despite the limitations of this systematic review, the results of the animal studies show that implants placed in healed sites with a flapless approach have better clinical parameters than the flapped procedure in a short-term follow-up. In human studies, there is no consensus about which technique offer better results in terms of clinical parameters. It should be taken into account that there are few studies comparing the effect of flapless vs. flapped surgery on clinical measurements around dental implants. Therefore, more research in humans is required in order to overcome the limitations and contrast these results.

## References

[1] Brånemark PI, Hansson BO, Adell R, Breine U, Lindström J, Hallén O (1977). Osseointegrated implants in the treatment of the edentulous jaw. Experience from a 10-year period. Scand J Plast Reconstr Surg Suppl.

[2] Albrektsson T, Brånemark PI, Hansson HA, Lindström J (1981). Osseointegrated titanium implants. Requirements for ensuring a long-lasting, direct bone-to-implant anchorage in man. Acta Orthop Scand.

[3] Esposito M, Murray-Curtis L, Grusovin MG, Coulthard P, Worthington HV (2007). Interventions for replacing missing teeth: different types of dental implants. Cochrane Database Syst Rev.

[4] Campelo LD, Camara JR (2002). Flapless implant surgery: A 10-year clinical retrospective analysis. Int J Oral Maxillofac Implants.

[5] Bayounis AM, Alzoman HA, Jansen JA, Babay N (2011). Healing of peri-implant tissues after flapless and flapped implant installation. J Clin Periodontol.

[6] Kim JI, Choi BH, Li J, Xuan F, Jeong SM (2009). Blood vessels of the peri-implant mucosa: a comparison between flap and flapless procedures. Oral Surg Oral Med Oral Pathol Oral Radiol Endod.

[7] Gomez-Roman G (2001). Influence of flap design on peri-implant interproximal crestal bone loss around single-tooth implants. Int J Oral Maxillofac Implants.

[8] Van der Zee E, Oosterveld P, Van Waas MA (2004). Effect of GBR and fixture installation on gingiva and bone levels at adjacent teeth. Clin Oral Implants Res.

[9] Wood DL, Hoag PM, Donnenfeld OW, Rosenfeld LD (1972). Alveolar crest reduction following full and partial thickness ﬂaps. J Periodontol.

[10] Nobuto T, Suwa F, Kono T, Taguchi Y, Takahashi T, Kanemura N (2005). Microvascular response in the periosteum following mucoperiosteal ﬂap surgery in dogs: angiogenesis and bone resorption and formation. J Periodontol.

[11] Kohler CA, Ramfjord SP (1960). Healing of gingival mucoperiostal ﬂaps. Oral Surg Oral Med Oral Pathol.

[12] Lobene R, Glickman I (1963). The response of alveolar bone to grinding with rotary diamond stones. J Periodontol.

[13] Caffesse RG, Ramfjord SP, Nasjleti CE (1968). Reverse bevel periodontal ﬂaps in monkeys. J Periodontol.

[14] Ledermann PD (1977). Complete denture provision of atrophic problem mandible with aid of CBS-implants. Quintessenz.

[15] Jeong SM, Choi BH, Li J, Kim HS, Ko CY, Jung JH (2007). Flapless implant surgery: an experimental study. Oral Surg Oral Med Oral Pathol Oral Radiol Endod.

[16] Tsoukaki M, Kalpidis CDR, Sakellari D, Tsalikis L, Mikrogiorgis G, Konstantinidis A (2013). Clinical, radiographic, microbiological, and immunological outcomes of flapped vs. flapless dental implants: a prospective randomized controlled clinical trial. Clin Oral Implants Res.

[17] Sunitha RV, Ramakrishnan T, Kumar S, Emmadi P (2008). Soft tissue preservation and crestal bone loss around single-tooth implants. J Oral Implantol.

[18] Becker W, Goldstein M, Becker BE, Sennerby L (2005). Minimally invasive ﬂapless implant surgery: a prospective multicenter study. Clin Implant Dent Relat Res.

[19] Rao W, Benzi R (2008). Single mandibular first molar implants with flapless guided surgery and immediate function: preliminary clinical and radiographic results of a prospective study. J Prosthet Dent.

[20] Hahn J (2000). Single-stage, immediate loading, and ﬂapless surgery. J Oral Implantol.

[21] Cloyd JS (2008). Flapless implant surgery for replacement of posterior teeth. Dent Today.

[22] Fortin T, Bosson JL, Isidori M, Blachet E (2006). Effect of ﬂapless surgery on pain experienced in implant placement using an image-guided system. Int J Oral Maxillofac Implants.

[23] Ramfjord SP, Costich ER (1968). Healing after exposure of periosteum on the alveolar process. J Periodontol.

[24] Cairo F, Pagliaro U, Nieri M (2008). Soft tissue management at implant sites. J Clin Periodontol.

[25] Jeong SM, Choi BH, Kim J, Xuan F, Lee DH, Mo DY (2011). A 1-year prospective clinical study of soft tissue conditions and marginal bone changes around dental implants after flapless implant surgery. Oral Surg Oral Med Oral Pathol Oral Radiol Endod.

[26] Sclar AG (2008). Guidelines for ﬂapless surgery. J Oral Maxillofac Surg.

[27] Lei Q, Chen J, Jiang J, Fu X, Lin H, Cai Z (2013). Comparison of soft tissue healing around implants in beagle dogs: flap surgery versus flapless surgery. Oral Surg Oral Med Oral Pathol Oral Radiol.

[28] Vlahovic Z, Markovic A, Golubovic M, Scepanovic M, Kalanovic M, Djinic A (2015). Histopathological comparative analysis of peri-implant soft tissue response after dental implant placement with flap and flapless surgical technique. Experimental study in pigs. Clin Oral Implants Res.

[29] Vohra F, Al-Kheraif AA, Almas K, Javed F (2015). Comparison of Crestal Bone Loss Around Dental Implants Placed in Healed Sites Using Flapped and Flapless Techniques: A Systematic Review. J Periodontol.

[30] Lin GH, Chan HL, Bashutski JD, Oh TJ, Wang HL (2014). The Effect of Flapless Surgery on Implant Survival and Marginal Bone Level: A Systematic Review and Meta-Analysis; J periodontol. J Periodontol.

[31] Liberati A, Altman DG, Tetzlaff J, Mulrow C, Gøtzsche PC, Ioannidis JP (2009). The PRISMA statement for reporting systematic reviews and meta-analyses of studies that evaluate health care interventions: explanation and elaboration. J Clin Epidemiol.

[32] Higgins JP, Altman DG, Gøtzsche PC, Jüni P, Moher D, Oxman AD (2011). Cochrane Bias Methods Group. Cochrane Statistical Methods Group. The Cochrane Collaboration's tool for assessing risk of bias in randomised trials. BMJ.

[33] Hooijmans CR, Rovers MM, de Vries RB, Leenaars M, Ritskes-Hoitinga M, Langendam MW (2014). SYRCLE's risk of bias tool for animal studies. BMC Med Res Methodol.

[34] Silness J, Löe H (1964). Periodontal disease in pregnancy. II. Correlation between oral hygiene and periodontal condition. Acta Odontol Scand.

[35] Löe H, Silness J (1963). Periodontal disease in pregnancy. I. Prevalence and severity. Acta Odontol Scand.

[36] Jemt T (1997). Regeneration of gingival papillae after single- implant treatment. Int J Periodontics Restorative Dent.

[37] Mombelli A, Lang NP (1994). Clinical parameters for the evaluation of dental implants. Periodontology 2000.

[38] Bashutski JD, Wang HL, Rudek I, Moreno I, Koticha T, Oh TJ (2013). Effect of flapless surgery on single-tooth implants in the esthetic zone: a randomized clinical trial. J periodontol.

[39] Al-Juboori MJ, bin Abdulrahaman S, Subramaniam R, Tawfiq OF (2012). Less morbidity with flapless implant. Dent Implantol Update.

[40] You TM, Choi BH, Li J, Xuan F, Jeong SM, Jang SO (2009). Morphogenesis of the peri-implant mucosa: a comparison between flap and flapless procedures in the canine mandible. Oral Surg Oral Med Oral Pathol Oral Radiol Endod.

[41] Gamborena I, Lee J, Fiorini T, Wenzel BA, Schüpbach P, Wikesjö UM (2015). Effect of Platform Shift/Switch and Concave Abutments on Crestal Bone Levels and Mucosal Profile following Flap and Flapless Implant Surgery. Clin Implant DentRelat Res.

[42] Wang F, Huang W, Zhang Z, Wang H, Monje A, Wu Y (2016). Minimally invasive flapless vs. flapped approach for single implant placement: a 2-year randomized controlled clinical trial. Clin Oral Implants Res.

